# Complete Response after Treatment with Neoadjuvant Chemoradiation with Prolonged Chemotherapy for Locally Advanced, Unresectable Adenocarcinoma of the Pancreas

**DOI:** 10.1155/2017/7834702

**Published:** 2017-03-08

**Authors:** Tiffany A. Pompa, William F. Morano, Chetan Jeurkar, Hui Li, Suganthi Soundararajan, Jaganmohan Poli, Wilbur B. Bowne, Michael Styler

**Affiliations:** ^1^Department of Medicine, Division of Hematology/Oncology, Drexel University College of Medicine, Philadelphia, PA 19102, USA; ^2^Department of Surgery, Division of Surgical Oncology, Drexel University College of Medicine, Philadelphia, PA 19102, USA; ^3^Department of Pathology & Laboratory Medicine, Drexel University College of Medicine, Philadelphia, PA 19102, USA; ^4^Department of Radiation Oncology, Drexel University College of Medicine, Philadelphia, PA 19102, USA

## Abstract

Surgery is the only chance for cure in pancreatic ductal adenocarcinoma. In unresectable, locally advanced pancreatic cancer (LAPC), the National Comprehensive Cancer Network (NCCN) suggests chemotherapy and consideration for radiation in cases of unresectable LAPC. Here we present a rare case of unresectable LAPC with a complete histopathological response after chemoradiation followed by surgical resection. A 54-year-old female presented to our clinic in December 2013 with complaints of abdominal pain and 30-pound weight loss. An MRI demonstrated a mass in the pancreatic body measuring 6.2 × 3.2 cm; biopsy revealed proven ductal adenocarcinoma. Due to splenic vein/artery and contiguous celiac artery encasement, she was deemed surgically unresectable. She was started on FOLFIRINOX therapy (three cycles), intensity modulated radiation to a dose of 54 Gy in 30 fractions concurrent with capecitabine, followed by FOLFIRI, and finally XELIRI. After 8 cycles of ongoing XELIRI completed in March 2015, restaging showed a remarkable decrease in tumor size, along with PET-CT revealing no FDG-avid uptake. She was reevaluated by surgery and taken for definitive resection. Histopathological evaluation demonstrated a complete R0 resection and no residual tumor. Based on this patient and literature review, this strategy demonstrates potential efficacy of neoadjuvant chemoradiation with prolonged chemotherapy, followed by surgery, which may improve outcomes in patients deemed previously unresectable.

## 1. Introduction

Pancreatic cancer is the fourth leading cause of cancer death in the United States with pancreatic ductal adenocarcinoma (PDAC), accounting for nearly 90% of the cases [1]. Surgery offers the best chance for cure with prolonged survival, yet only 15–20% of patients present as candidates for resection at diagnosis [2, 3]. Nonmetastatic, locally advanced, pancreatic cancer (LAPC) is observed in about 40% of patients upon presentation thus posing a difficult challenge for the oncologist.

The most important aspect after diagnosis of PDAC is determining resectability of the tumor. Dedicated radiographic imaging of PDAC serves as a window for an experienced radiologist and pancreatic surgeon to determine resectability. The recommended imaging modality for visualization of a pancreatic tumor and surrounding structures is multidetector computed tomography (MDCT) which uses thin-sliced, incremental vascular phases and parenchymal contrast to assess advanced disease [4]. These images are best obtained prior to interventions such as biopsy or stent placement, as these can limit accuracy of interpretation [5]. Magnetic resonance imaging may be used to further define local and distant extent of disease.

As per the National Comprehensive Cancer Network (NCCN) guidelines, presentation of PDAC is divided into resectable, borderline resectable, and unresectable (LAPC and metastatic) disease, with distinctions for pancreatic anatomy, as well as venous and arterial involvement [6, 7]. Traditionally, the goals of care for patients with unresectable disease have been to extend survival and limit symptomatology with palliative intent using chemotherapy and/or radiation. More recently, focus has shifted toward the use of chemoradiation to downstage borderline resectable disease, allowing for resection [8–10].

A complete pathological response describes the presence of neoplastic tissue without viable invasive cancer cells within the specimen [11]. In pancreatic cancer, a complete pathologic response is associated with improved survival, yet a complete pathologic response following neoadjuvant therapy in LAPC is rarely reported [11, 12]. We present a case of a patient with a locally advanced, initially unresectable PDAC of the body and tail, who underwent neoadjuvant chemoradiation, with prolonged chemotherapy, followed by R0 radical resection that included subtotal pancreatectomy, splenectomy, and left adrenalectomy with no residual tumor identified in the pathologic specimen.

## 2. Case Report

**Figure 1:**
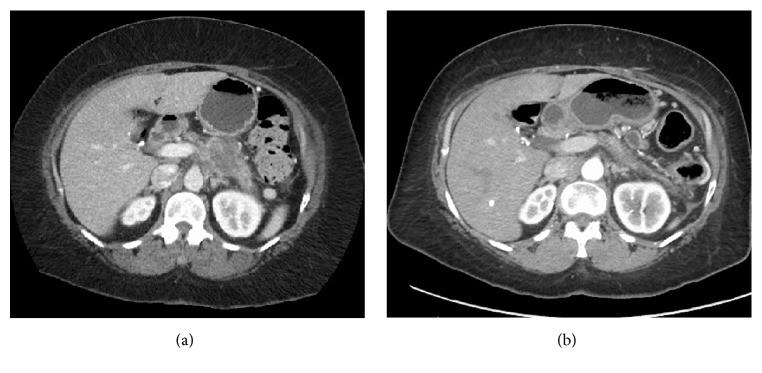
(a) Axial CT demonstrating large mass in body/tail of pancreas before neoadjuvant therapy. (b) Axial CT demonstrating tumor response after neoadjuvant therapy.

A 54-year-old female presented in December 2013 with abdominal pain and 30-pound weight loss. A pancreatic mass was identified on routine cross-sectional imaging. CT with pancreatic protocol revealed an ill-defined soft tissue density in the region of the pancreatic body with proximal pancreatic ductal dilation central necrosis and obliteration of the splenic vein and encasement of the splenic and celiac arteries (Figure 1(a)). An MRI confirmed a soft tissue mass in the body of the pancreas, measuring 6.2 × 3.2 cm. An endoscopic ultrasound and fine-needle aspiration of the mass revealed PDAC and confirmed vascular encasement. Her initial CA 19-9 was elevated at 531. She was then evaluated by the surgical oncology service that determined the lesion was unresectable due to locally advanced extension of tumor with vascular involvement.

**Figure 2:**
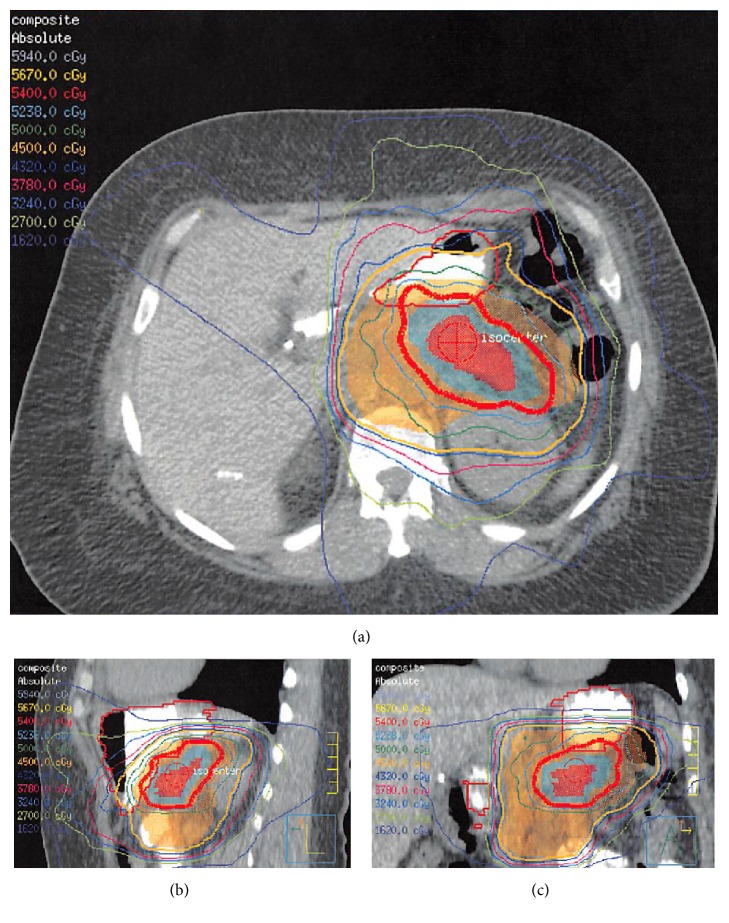
Axial, sagittal, and coronal isodose distribution of IMRT plan to 54 Gy in 30 fractions.

The patient had an optimal ECOG performance status despite her significant weight loss. She began neoadjuvant chemotherapy with three cycles of FOLFIRINOX (5-FU, leucovorin, irinotecan, and oxaliplatin) followed by concurrent intensity modulated radiation therapy (IMRT) to a dose of 54 Gy in 30 fractions (Figure 2) and capecitabine, which was tolerated well, except for development of mild peripheral neuropathy [13]. A repeat CT scan of the abdomen in July 2014, approximately 6 weeks after completing concurrent chemotherapy and radiation, showed a reduction in the size of her tumor to 3.7 × 2.7 × 3.9 cm, although there was persistent encasement of the celiac and splenic arteries.

Chemotherapy with FOLFIRI (5-FU, leucovorin and irinotecan) was started in July 2014 [14]. However, to obviate the need for central venous catheter placement, this regimen was later switched to XELIRI (capecitabine and irinotecan) beginning with cycle two [15]. Repeat CT imaging of the abdomen in December 2014, after her fifth cycle of XELIRI, revealed mild decrease in the size of the pancreatic mass but persistent peripancreatic soft tissue involvement and encasement of the celiac and splenic arteries.

**Figure 3:**
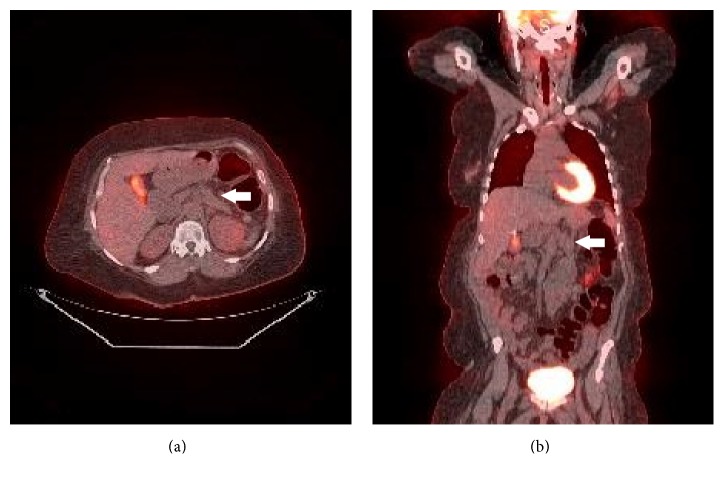
PET-CT ((a) axial, (b) coronal) images showing lack of FDG-avid uptake in pancreas after neoadjuvant chemoradiation.

We performed a restaging PET-CT in January 2015, eight months after initiation of therapy, which showed no FDG-avid uptake in the pancreas or the surrounding structures (Figure 3). Minimal stranding in the peripancreatic tissue was believed to be treatment-related fibrosis replacing tumor. She received a total of eight cycles of XELIRI, completed in March 2015. At that time, repeat CT imaging showed decrease in the size of her mass and persistent splenic artery encasement but minimal involvement of the celiac artery and findings consistent with possible radiation-induced fibrosis (Figure 1(b)). Additionally, at that time, her CA 19-9 was normal (14 U/mL).

**Figure 4:**
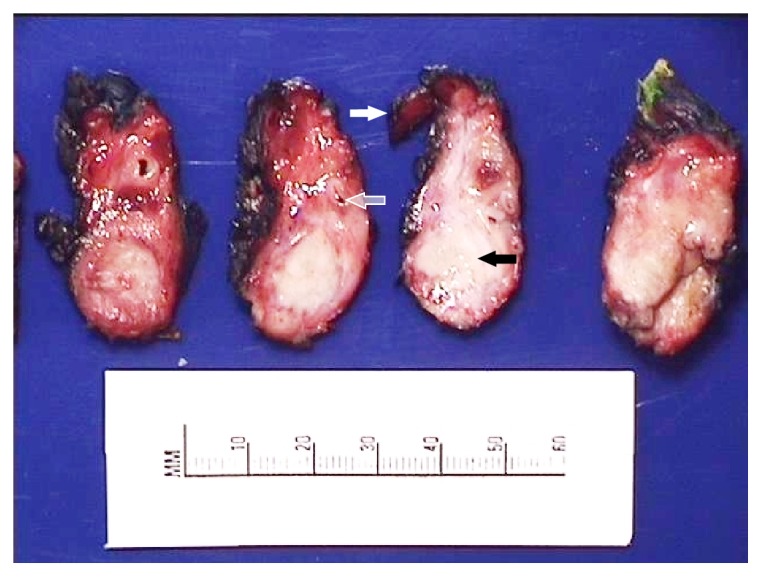
Cross sections of gross pathologic specimen. Black arrow points to fibrosis within the body of the resected pancreas. White arrow points to resected adrenal gland. Translucent arrow denotes the splenic artery encased in fibrosis.

**Figure 5:**
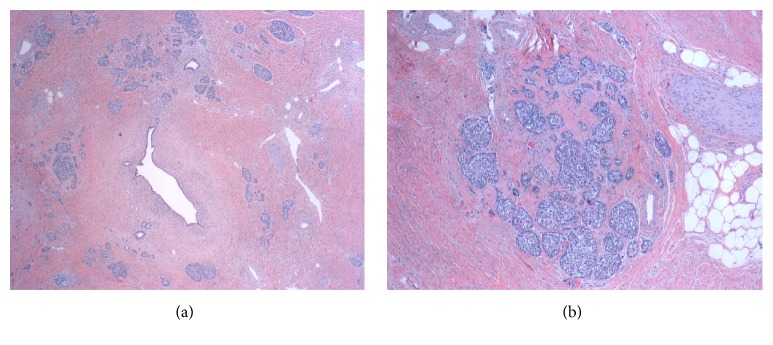
(a) H&E sections demonstrating pancreatic tissue with dense fibrosis, residual ducts, and islet cells. No evidence of residual carcinoma. (b) Cluster of islets of Langerhans within the fibrotic tissue.

Given the negative FDG-avid uptake on PET-CT and significantly improved CT, the patient was considered for surgical resection. After discussion at our multidisciplinary tumor conference, she was taken for radical antegrade modular pancreatosplenectomy in April 2015 [16, 17]. Initially, a diagnostic laparoscopy was performed, which did not demonstrate evidence of disseminated or peritoneal metastasis. The procedure continued with a left subcostal incision, which was extended to the right side. After reflection of the stomach superiorly and transverse colon inferiorly, a large degree of dense-appearing fibrosis was found obscuring the ventral surface of the pancreas with anterior and posterior extension to the spleen and left adrenal gland, respectively. The posterior surface of the stomach did not show any involvement. The body and tail of the pancreas and spleen were subsequently resected en bloc with Gerota's fascia and left adrenal gland. The celiac artery was skeletonized and appeared to have no residual disease. The procedure continued to conclusion without complications and with approximate estimated blood loss of 500 mL. Her postoperative course was complicated by pneumonia. The patient was discharged home on postoperative day 16. During the immediate 60-day postoperative period, there was no surgery-related morbidity. Histopathological analysis revealed an R0 resection with no residual tumor, but extensive fibrosis was found throughout the surgical specimen to the margins (Figures 4 and 5). The ten lymph nodes evaluated were negative. Ten months following resection, a small focus of local disease recurrence was detected on PET-CT and confirmed by fine-needle aspiration. The patient was subsequently treated with proton radiation therapy at an outside hospital and followed for three months until death from a treatment-related complication.

## 3. Discussion

The goals of treatment for patients with LAPC are predominantly palliation and extending survival. Patients with borderline disease may benefit from neoadjuvant chemoradiation, increasing the potential for operative resection. This neoadjuvant treatment paradigm was originally described in a case series of patients with borderline disease from Varadhachary et al. in 2006 [18]. Evans et al. later published an expert consensus statement in 2009, which recommended an aggressive approach for this patient population, in collaboration with medical and radiation oncologists [19]. A similar strategy may prove applicable for unresectable LAPC.

In 2013, Heinemann et al. reported an overall resection rate after neoadjuvant therapy in LAPC of 33%, 79% of which had negative resection margins. Patients that remained unresectable had recorded median survival of 10.2 months, while the 33% of patients that underwent resection had a median overall survival of 20.5 months [6]. In this report, up to one-third of patients with initially unresectable LAPC became resectable after neoadjuvant therapy with chemotherapy, radiotherapy, or a combination of the two [6]. Importantly, if R0 resection could be achieved, overall survival was similar to those with primary resectable disease [6, 20].

Current treatment approaches for unresectable LAPC involve chemotherapy and/or radiation [9]. Treatment goals include controlling the spread of tumor, reducing metastatic potential, and downstaging of tumor status to allow for possible surgical resection. Chemotherapeutic regimens in LAPC are extrapolated from their use in metastatic disease. The Accord 11 trial demonstrated FOLFIRINOX to be superior to single agent gemcitabine in patients with metastatic disease. This study showed that a combination of 5-fluorouracil, irinotecan, and oxaliplatin (FOLFIRINOX) produced increased response rates, progression-free survival, and overall survival (6.8 versus 11.1 months, *p* < 0.001) compared to standard monotherapy with gemcitabine [13]. Based on this response rate, FOLFIRINOX followed by radiation became the rational treatment option as neoadjuvant therapy in these patients, like ours, with unresectable LAPC. Hosein et al. subsequently reported an R0 resection rate of 44% in patients who received FOLFIRINOX as neoadjuvant therapy [21]. Moreover, a recent observational study including 101 patients with locally advanced, unresectable disease treated with FOLFIRINOX as induction therapy showed that 29% of patients had a reduction in tumor size of and half of those patients underwent resection with 55% undergoing an R0 resection [10].

Radiographic criteria to determine response to neoadjuvant therapy remain equivocal for borderline and more so for LAPC [22]. In our patient, evaluation of treatment efficacy was determined by RECIST Criteria with a CT dedicated pancreatic protocol. PET-CT imaging was also used to measure associated metabolic, tumor-related activity, which was not detected [23]. In monitoring treatment efficacy, Picchio et al. found that PET-CT can detect a metabolic response to treatment not identified by CT, while Chang et al. found PET-CT scanning to be a more effective method for evaluating tumor response than conventional CT scanning [24, 25]. There have also been noted instances where patients were spared unnecessary pancreaticoduodenectomy after “occult” metastases were revealed on PET imaging [24]. Nevertheless, the role of PET-CT in diagnosis and treatment response for PDAC remains controversial. NCCN currently recommends not to substitute a PET-CT for high quality standard CT evaluation in any stage of pancreatic cancer [7].

Challenges for evaluating response to neoadjuvant therapy include radiographic underestimation of treatment-related effects, such as peripancreatic inflammation and fibrosis [26]. Ferrone et al. recently published the largest study of LAPC patients who underwent neoadjuvant treatment and surgical resection. This study concluded that, after neoadjuvant FOLFIRINOX and RT (in most patients), imaging no longer accurately predicted patients for resectability. Among 40 patients undergoing resection, 19 were deemed unresectable by imaging, yet there was a 92% R0 resection rate. This study used surgical exploration with biopsies prior to proceeding to resection [8].

Neoadjuvant therapy-induced fibrosis often masquerades as residual, posttreatment tumor. Sasson et al. performed a retrospective review of 116 patients who underwent resection for PDAC following neoadjuvant therapy, demonstrating 56% mean fibrosis level in surgical specimens. Patients that received neoadjuvant therapy, as well as those with negative margins and lymph nodes, had higher levels of fibrosis than those patients with no preoperative treatment. Patients undergoing neoadjuvant therapy had higher pathological curative resection rates and median survival (23 mo versus 16 mo) [27, 28]. As recently shown, patients who undergo a prolonged time interval after neoadjuvant chemoradiation with ongoing chemotherapy are more likely to have an improved pathological response at time of surgical resection, which is associated with improved median overall survival; an apparent benefit observed in this case report (30-month survival) [29].

The use of combined neoadjuvant treatment with chemotherapy and/or radiation increases median survival for patients with locally advanced cancers to approximately 9 to 13 months but rarely results in long-term survival [30]. Despite this, the percentage of approximately 17,000 new LAPC cases diagnosed each year, considered for neoadjuvant therapy, and followed by resection in the United States remains small [6]. Reasons for this include indeterminate resectability of vascular involvement, patient comorbidities, oncology/community bias, and insufficient surgical expertise. The NCCN guidelines now recommend that patients with unresectable LAPC that demonstrate a significant response to therapy be considered for resection [7].

## 4. Conclusion

In our patient after neoadjuvant chemoradiation, with prolonged time interval and ongoing chemotherapy, a notable change in disease-status on imaging and normalization of surrogate tumor markers warranted an aggressive approach to therapy. A complete pathological response was determined, in this patient, after resection. With recent advances in neoadjuvant therapies (e.g., FOLFIRINOX, IMRT) and comprehensive multidisciplinary approach, more patients with unresectable LAPC may become candidates for curative resection. Pancreatic cancer is projected to become the second leading cause of cancer death in the US by 2030; treatment strategies to increase resectability are clearly needed [31]. This approach should be considered in carefully selected patients and validated by future studies in this patient population.
